# Level of agreement between two asthma control questionnaires in children and adolescents

**DOI:** 10.36416/1806-3756/e20240407

**Published:** 2025-05-21

**Authors:** Cláudio Luiz Castro Gomes de Amorim, Karina Couto Furlanetto, Thaila Corsi, Fabio Pitta

**Affiliations:** 1. Programa de Pós-Graduação em Ciências da Saúde, Universidade Estadual de Londrina, Londrina (PR) Brasil.; 2. Laboratório de Pesquisa em Fisioterapia Pulmonar, Departamento de Fisioterapia, Universidade Estadual de Londrina, Londrina (PR) Brasil.; 3. Centro de Pesquisa e Pós-Graduação, Universidade Pitágoras/Universidade Norte do Paraná - UNOPAR - Londrina (PR) Brasil.

**Keywords:** Asthma, Pediatrics, Surveys and questionnaires, Therapeutics

## Abstract

**Objective:**

To investigate the level of agreement between the GINA questionnaire and the Asthma Control Test (ACT) in children and adolescents with asthma, as well as to compare the clinical, laboratory, and spirometric characteristics of patients with controlled and uncontrolled asthma as assessed by the two questionnaires.

**Methods:**

Children and adolescents with asthma had their level of asthma control cross-sectionally assessed by the GINA questionnaire and the ACT, the Childhood ACT (C-ACT) being used for the children in the sample. The participating patients also underwent spirometry, clinical assessment (for passive smoking, asthma severity, medication use, and type of inhaler device used), and laboratory measurements of vitamin D levels, blood eosinophils, and total IgE.

**Results:**

A total of 76 patients were assessed. Of those, 62% were male, and the mean age was 10 [9-12] years. In addition, 42% and 20% were classified as having uncontrolled asthma by the GINA questionnaire and the C-ACT/ACT, respectively. There was moderate agreement between the two questionnaires regarding identification of controlled and uncontrolled asthma (k = 0.505; p < 0,0001). The patients classified as having uncontrolled asthma by the GINA questionnaire had worse results in terms of daily inhaled corticosteroid dose, asthma severity, passive smoking, and spirometric parameters (FVC, FEV_1_, and FEF_25-75%_). However, there was no significant difference between patients classified as having controlled or uncontrolled asthma by the C-ACT/ACT.

**Conclusions:**

In children and adolescents with asthma, the level of agreement between the GINA questionnaire and the C-ACT/ACT appears to be moderate. The GINA questionnaire appears to have a stronger association with functional parameters, indicating better clinical assessment with less underreporting of uncontrolled asthma.

## INTRODUCTION

Asthma is a chronic, heterogeneous disease characterized by airway inflammation. It is the most common chronic disease among children and adolescents,^1^ and remains a public health issue despite efforts to reduce hospitalizations and mortality. In children and adolescents, the prevalence of asthma is increasing, posing a burden for patients and their parents.^2^

The diagnosis of asthma is based on identification of a recurring pattern of wheezing, dyspnea, chest tightness, and/or cough that may improve with the use of bronchodilators, as well as on documentation of airflow limitation, whenever possible.^2^ There is a wide range of therapeutic medications, particularly inhaled corticosteroids (ICSs), which may be combined with long-acting b_2_ agonists (LABAs).^2^

Once asthma medication treatment begins, patient symptoms must be periodically reassessed in order to maintain symptom control, thereby reducing the risk of exacerbations and mortality. To achieve this goal, clinical questionnaires are used in order to evaluate asthma control and adjust asthma medication therapy.^2^ Among the questionnaires assessing asthma control, the most well-known are the GINA questionnaire^2^ and the Asthma Control Test (ACT), with the Childhood Asthma Control Test (C-ACT) being used for children^1^ ^,^ ^3^ and the ACT being used for adolescents.^4^ ^,^ ^5^ Each questionnaire is applied differently. The GINA questionnaire is easy to apply, being administered verbally by the physician to the patient or their legal guardian and addressing aspects such as symptom frequency, medication use, and limitations in daily activities through four yes or no questions.^6^ The C-ACT/ACT are printed questionnaires answered by the patient and their legal guardian (in the case of the C-ACT)^3^ or by the patient only (in the case of the ACT),^5^ with each question being assigned a score that is summed up (the maximum being 27 for the C-ACT and 25 for the ACT). 

These questionnaires for assessing asthma control are widely used and of great importance because patients and physicians tend to overestimate the level of disease control and the improvement achieved with treatment.^6^ However, neither questionnaire includes lung function parameters. Although this simplifies their application, it also means that they fail to provide a functional evaluation of patients with asthma by failing to identify changes in lung function.^7^

Although the GINA questionnaire and the C-ACT/ACT are widely used, it is yet unknown whether their results are consistent or even interchangeable. Therefore, the objective of the present study was to assess the level of agreement between the GINA questionnaire and the C-ACT/ACT in children and adolescents with asthma, as well as to compare the clinical, laboratory, and spirometric characteristics of patients with controlled and uncontrolled asthma as assessed by the two questionnaires. 

## METHODS

The present study was conducted at the Pediatric Pulmonology Outpatient Clinic of the University Hospital of the State University at Londrina, located in the city of Londrina, Brazil. This was a primary study in which an analytical cross-sectional design was used. 

A convenience sample of patients treated at the outpatient clinic between May of 2019 and August of 2023 were consecutively recruited to participate in the study. All participants were assessed at a single time point, undergoing clinical, laboratory, and spirometric evaluations. All of the patients who met the inclusion criteria and agreed to participate in the study were consecutively included and evaluated. The legal guardians of all participating patients gave written informed consent. The study was approved by the Human Research Ethics Committee of State University at Londrina (Protocol no. 3.093.047/2018). 

The inclusion criteria were as follows: being in the 6- to 14-year age bracket; being followed at the Pediatric Pulmonology Outpatient Clinic of the University Hospital of the State University at Londrina; having received a clinical diagnosis of asthma in accordance with the GINA criteria^2^; currently using an ICS (beclomethasone, budesonide, or fluticasone), with or without a LABA, with no restrictions on the duration of use; being clinically stable, without the need for oral corticosteroids in the last month because of asthma exacerbation; having no other lung diseases (e.g., chronic lung disease of prematurity, obliterative bronchiolitis, bronchiectasis, and cystic fibrosis); having no signs or symptoms of gastroesophageal reflux disease or dysphagia; and having used an antiparasitic drug in the last 12 months. The exclusion criteria were as follows: having taken vitamin D supplementation (because vitamin D has an impact on modulation of inflammation and, consequently, on asthma control); and having experienced asthma exacerbations requiring hospitalization for more than one day and/or the use of oral corticosteroids in the last month. 

The participating patients underwent assessment of asthma severity and control level with the GINA questionnaire^2^ and the C-ACT/ACT.^1^ ^,^ ^3^ ^-^ ^5^ They also underwent spirometry and laboratory measurements of blood eosinophils and total IgE. 

The GINA asthma control questionnaire consists of 4 yes or no questions regarding the past four weeks. The questions are as follows: 1) Asthma-like symptoms during the day for more than a few minutes, more than once a week?; 2) Any activity limitation due to asthma?; 3) Need for reliever medication more than once a week?; and 4) Any nighttime awakening or cough due to asthma? If all responses are “no,” the patient is classified as having controlled asthma; if 1 or 2 questions have an affirmative response, the classification is partially controlled; if 3 or 4 responses are affirmative, the disease is classified as uncontrolled.^2^ The questions on the GINA questionnaire were asked directly to the patients, who were however assisted by their parents/legal guardians/companions whenever needed; they could intervene in case there were any questions or difficulties. For analysis purposes, the patients in the present study were classified as having controlled or uncontrolled asthma, with the latter group including patients with partially controlled and uncontrolled asthma. 

The C-ACT and the ACT were applied in accordance with patient age. For children < 12 years of age, the C-ACT was used. The C-ACT consists of seven questions, four of which are answered by the patients themselves and three of which are answered by their parents or legal guardians, with a minimum score of 0 and a maximum score of 27. A higher score translates to a better asthma control. Scores between 20 and 27 classify patients as having controlled asthma; scores between 15 and 19 classify patients as having partially controlled asthma; and scores between 0 and 14 classify patients as having uncontrolled asthma.^3^ Children ≥ 12 years of age completed the ACT, which consists of five questions. Each question is scored on a scale of 1 to 5 points; therefore, the minimum total score is 5, and the maximum total score is 25. A higher score translates to a better asthma control. Scores between 20 and 25 classify patients as having controlled asthma; scores between 16 and 19 classify patients as having partially controlled asthma; and scores between 5 and 15 classify patients as having uncontrolled asthma.^2^ For analysis purposes, children and adolescents were classified as having controlled or uncontrolled asthma, with the latter group including patients with partially controlled and uncontrolled asthma. In summary, the patients in the present study were divided into two groups based on asthma control (controlled or uncontrolled) as assessed by the GINA questionnaire and the C-ACT/ACT. For the GINA questionnaire, patients in the controlled asthma group (the GINA controlled asthma group) were characterized by answering “no” to all four questions, whereas those in the uncontrolled asthma group (the GINA uncontrolled asthma group) had at least one affirmative response.^2^ For the C-ACT/ACT, controlled asthma (the ACT controlled asthma group) was characterized by a score of 20 or higher, whereas uncontrolled asthma (the ACT uncontrolled asthma group) had a score of 19 or lower.^3^ ^,^ ^4^

All participating patients underwent spirometry with a MiniSpir spirometer (MIR, Rome, Italy) and the WinspiroPRO software, version 5.9, the technique recommended by the American Thoracic Society being used.^8^ Reference values for the Brazilian population^9^ were used. All analyses utilized pre-bronchodilator spirometry results and the pre- and post-bronchodilator variation. Obstructive ventilatory disorder was defined by a reduction in the FEV_1_/FVC ratio below the 5th percentile of the predicted value (lower limit).^10^

With regard to the laboratory tests for sample characterization, immunoturbidimetry (Architect c8000; Abbott Laboratories, Abbott Park, IL, USA) was used for IgE analysis and quantification. An automated method was used for serum eosinophils, as described elsewhere.^11^

The inhalers and medications used by the participating patients were also recorded. Inhaler devices included metered dose inhalers and dry powder inhalers such as Turbuhaler^®^ and Aerolizer^®^. Additionally, the sample included patients using beclomethasone and budesonide. For dose analysis, the corresponding budesonide dose was used, as recommended by GINA,^2^ varying according to patient age (6-11 years of age and > 12 years of age). ICS doses were based on budesonide and were categorized as low, medium, or high.^2^ For the age group of 6-11 years, a budesonide dose of 100-200 µg/day was defined as low; a budesonide dose > 200 ≤ 400 µg/day was defined as medium; and a budesonide dose > 400 µg/per day was defined as high.^2^ For patients > 12 years of age, a dose of 200-400 µg/day was defined as low; a dose > 400 ≤ 800 µg/day was defined as medium; and a dose > 800 µg/day was defined as high.^2^ Formoterol was the only LABA used in association with budesonide. Asthma severity was classified as mild, moderate, or severe on the basis of the GINA stepwise system, as follows: mild (for patients receiving GINA step 1 or 2 treatment); moderate (for patients receiving GINA step 3 treatment); and severe (for patients receiving GINA step 4 or 5 treatment).^2^

In the statistical analysis, the Shapiro-Wilk test was used in order to analyze the normality of data distribution. Normally distributed data were described as mean and standard deviation, and non-normally distributed data were described as median and interquartile range. Comparisons of continuous variables were performed with the unpaired Student’s t-test (for parametric variables) or the Mann-Whitney test (for nonparametric variables), whereas categorical variables were analyzed with the chi-square test and reported as absolute and relative values (proportions). The level of agreement between the controlled and uncontrolled asthma groups as assessed by the GINA questionnaire or the C-ACT/ACT was determined by calculating the kappa statistic, as follows: < 0, no agreement; 0-0.2, very low agreement; 0.2-0.4, fair agreement; 0.4-0.6, moderate agreement; 0.6-0.8, substantial agreement; and 0.8-1, excellent agreement.^12^ The Fisher’s exact test was used to identify statistically significant difference between the proportion of individuals classified as having controlled asthma according to the two classifications. The strength of association between the GINA questionnaire and the C-ACT/ACT was determined by calculating Cramér’s V, as follows: 0-0.10, negligible association; 0.11-0.20, weak association; 0.21-0.40, moderate association; 0.41-0.60, relatively strong association; 0.61-0.80, strong association; 0.81-0.99, very strong association; and 1, perfect association.^13^ All statistical analyses were performed with the IBM SPSS Statistics software package, version 25.0 (IBM Corporation, Armonk, NY, USA), and the level of significance was set at p < 0.05. 

## RESULTS

**Figure 1:**
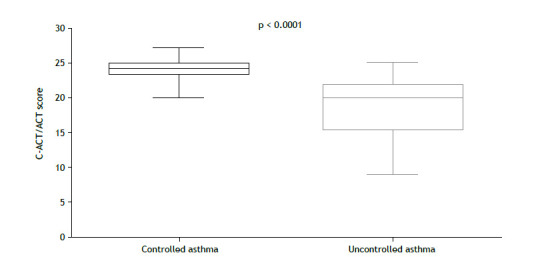
Comparison of Childhood Asthma Control Test/Asthma Control Test (C-ACT/ACT) scores for controlled and uncontrolled asthma as assessed by the GINA questionnaire.

Seventy-six patients were included in the present study, with no exclusions. The baseline characteristics of the participating patients are detailed in Table 1. The median age of the patients was 10 [9-12] years, and the median BMI was 18 [15-22] kg/m^2^. The C-ACT/ACT scores were significantly lower in the GINA uncontrolled asthma group than in the GINA controlled asthma group (20 [15-22] points vs. 24 [23-25] points; Figure 1). 

**Table 1:** Clinical, spirometric, and laboratory characteristics of the study sample (N = 76).^a^

Variable	
Sex, male/female	47/29 (62/38)
Age, years	10 [9-12]
Race, White/Black	61/15 (80/20)
BMI, kg/m^2^	18 [15-22]
GINA, uncontrolled asthma	32 (42)
C-ACT/ACT, uncontrolled asthma	15 (20)
Inhaler device	
Metered dose inhaler	29 (38)
Turbuhaler^®^	14 (19)
Aerolizer^®^	33 (43)
Medication	
Beclomethasone	1 (1)
Budesonide	25 (33)
Formoterol + budesonide	50 (66)
Daily dose, µg	405 ± 148
Dose load	
Low	20 (26)
Medium	49 (65)
High	7 (9)
GINA treatment classification	
Step 1 (mild asthma)	0 (0)
Step 2 (mild asthma)	9 (12)
Step 3 (moderate asthma)	25 (33)
Step 4 (severe asthma)	38 (50)
Step 5 (severe asthma)	4 (5)
Asthma severity	
Mild	9 (12)
Moderate	25 (33)
Severe	42 (55)
Passive smoking	35 (46)
Spirometry	
FVC, L	2.45 ± 0.68
FVC, % predicted	97 ± 14
ΔFVC (pre- and post-BD), %	3.32 [−0.57 to 8.43]
FEV_1_, L	2.01 ± 0.59
FEV_1_, % predicted	90 ± 18
ΔFEV_1_ (pre- and post-BD), %	6.61 [3.05-14.22]
FEV_1_/FVC	83 [78-88]
FEV_1_/FVC, % predicted	95 [88-99]
ΔFEV_1_/FVC (pre- and post-BD), %	3.24 [0.89-7.43]
FEF_25-75%_, L/s	2.12 ± 0.87
FEF_25-75%_, % predicted	79 ± 29
ΔFEF_25-75%_ (pre- and post-BD), %	16 [5-33]
Laboratory test results	
IgE, IU/mL*	519 [250-1,281]
Eosinophils, cells/µL*	588 [340-814]

C-ACT: Childhood Asthma Control Test; ACT: Asthma Control Test; and BD: bronchodilator. ^a^Data presented as n (%), mean ± SD, or median [IQR]. *n = 68.

**Figure 2:**
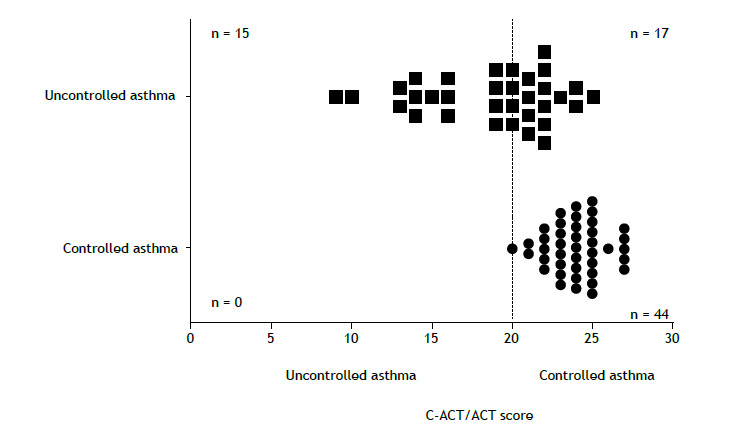
Controlled and uncontrolled asthma groups as assessed by the Childhood Asthma Control Test/Asthma Control Test (C-ACT/ACT) and the GINA questionnaire.

There was moderate agreement (κ = 0.505; p < 0.0001) between the controlled and uncontrolled asthma groups as assessed by the GINA questionnaire and the C-ACT/ACT, with good sensitivity (0.72) and excellent specificity (1.00). All patients classified as having controlled asthma by the GINA questionnaire were also classified as having controlled asthma by the C-ACT/ACT; however, not all patients classified as having uncontrolled asthma by the GINA questionnaire were also classified as having uncontrolled asthma by the C-ACT/ACT, with a discrepancy of 22% of the sample. Additionally, 58% of the sample was categorized as having controlled asthma by the GINA questionnaire, whereas 80% were categorized as having controlled asthma by the C-ACT/ACT, with a significant difference between the two evaluation methods (Fisher’s exact test, p < 0.0001). Nevertheless, there was a relatively strong association between the GINA and C-ACT/ACT classifications (Cramér’s V = 0.581; p < 0.0001; Figure 2). 

All comparisons between individuals with controlled and uncontrolled asthma as assessed by the GINA questionnaire and the C-ACT/ACT are shown in Table 2. When we compared the GINA controlled asthma and uncontrolled asthma groups, we found a significant difference in variables such as type of inhaler device, daily ICS dose, GINA treatment step, asthma severity, passive smoking, and spirometric parameters (FVC, FEV_1_, and FEF_25-75%_). However, we found no significant differences between the ACT controlled asthma and uncontrolled asthma groups. 

**Table 2:** Comparisons between children and adolescents with controlled and uncontrolled asthma as assessed by the GINA questionnaire and the Childhood Asthma Control Test/Asthma Control Test.^a^

	GINA questionnaire N = 76				C-ACT/ACT N = 76		
Variable	Controlled asthma (n = 44)	Uncontrolled asthma (n = 32)	p	Controlled asthma (n = 61)	Uncontrolled asthma (n = 15)	p
Sex, M/F	30 (68)/14 (32)	17 (53)/15 (47)	0.182	39 (64)/22 (36)	8 (53)/7 (47)	0.645
Age, years	10.2 ± 2.3	10 ± 2.1	0.773	10.2 ± 2.2	9.7 ± 2.1	0.457
BMI, kg/m^2^	18 [16-23]	18 [15-21]	0.388	18 [16-22]	20 [14-21]	0.700
Inhaler device			0.013			0.376
Metered dose inhaler	12 (27)	17 (53)		23 (38)	6 (40)	
Turbuhaler^®^	8 (18)	6 (19)		9 (15)	5 (33)	
Aerolizer^®^	24 (55)	9 (28)		29 (48)	4 (27)	
Medication			0.124			0.187
Beclomethasone	1 (2)	0 (0)		1 (2)	0 (0)	
Budesonide	17 (39)	8 (25)		22 (36)	3 (20)	
Budesonide + formoterol	26 (59)	24 (75)		38 (62)	12 (80)	
Daily dose, µg	372 ± 104	445 ± 173	0.049	386 ± 123	470 ± 191	0.075
Dose load			0.071			0.198
Low	14 (32)	6 (19)		17 (28)	3 (20)	
Medium	28 (63)	21 (65)		40 (65)	9 (60)	
High	2 (5)	5 (16)		4 (7)	3 (20)	
GINA treatment classification			0.026			0.080
Step 1 (mild asthma)	0 (0)	0 (0)		0 (0)	0 (0)	
Step 2 (mild asthma)	6 (14)	3 (9)		8 (13)	1 (7)	
Step 3 (moderate asthma)	19 (43)	6 (19)		22 (36)	3 (20)	
Step 4 (severe asthma)	18 (41)	20 (63)		29 (48)	9 (60)	
Step 5 (severe asthma)	1 (2)	3 (9)		2 (3)	2 (13)	
Asthma severity			0.043	3 [2-3]	3 [2-3]	0.128
Mild	6 (14)	3 (9)		8 (13)	1 (7)	
Moderate	19 (43)	6 (19)		22 (36)	3 (20)	
Severe	19 (43)	23 (72)		31 (51)	11 (7)	
Passive smoking			0.025			0.314
No	33 (75)	16 (50)		41 (67)	8 (53)	
Yes	11 (25)	16 (50)		20 (33)	7 (47)	
Spirometry						
FVC, L	2.53 ± 0.65	2.33 ± 0.71	0.214	2.49 ± 0.64	2.26 ± 0.80	0.259
FVC, % predicted	100 ± 14	93 ± 14	0.047	97 ± 14	95 ± 16	0.639
ΔFVC (pre- and post-BD), %	2 [−1 to 8]	5 [0-9]	0.391	3 [−1 to 8]	4 [0-9]	0.845
FEV_1_, L	2.09 ± 0.58	1.88 ± 0.59	0.127	2.04 ± 0.56	1.83 ± 0.67	0.218
FEV_1_, % predicted	94 ± 18	85 ± 17	0.040	91 ± 17	87 ± 21	0.500
ΔFEV_1_ (pre- and post-BD), %	6 [3-11]	8 [2-16]	0.288	7 [4-15]	6 [1-14]	0.422
FEV_1_/FVC	84 [78-89]	82 [76-87]	0.374	81 [78-88]	82 [74-92]	0.615
FEV_1_/FVC, % predicted	95 [89-100]	93 [86-98]	0.333	95 [89-100]	94 [81-100]	0.485
ΔFEV_1_/FVC (pre- and post-BD), %	3 [1-8]	5 [2-7]	0.222	3 [1-7]	2 [−1 to 10]	0.980
FEF_25-75%_, L/s	2.20 [1.64-2.92]	2.02 [1.43-2.42]	0.092	2.17 [1.5-72.74]	2.16 [0.87-2.46]	0.285
FEF_25-75%_, % predicted	89 [61-112]	71 [55-86]	0.028	82 [63-103]	78 [41-99]	0.312
ΔFEF_25-75%_ (pre- and post-BD), %	16 [1-29]	16 (8-40]	0.446	17 [7-32]	9 [2-69]	0.382

C-ACT: Childhood Asthma Control Test; ACT: Asthma Control Test; and BD: bronchodilator. ^a^Data presented as n (%), mean ± SD, or median [IQR].

## DISCUSSION

The present study identified significant differences in the proportions of children classified as having controlled or uncontrolled asthma by two different questionnaires assessing disease severity. Specifically, the C-ACT/ACT identified a smaller proportion of children with uncontrolled asthma than did the GINA questionnaire, despite a strong association and moderate agreement between the two questionnaires. Additionally, among the children with uncontrolled asthma as assessed by the GINA questionnaire, metered dose inhalers were the most commonly used inhaler devices; GINA treatment step 4 (severe asthma)^2^ was the most commonly given treatment; passive smoking was highly prevalent; and spirometric values were worse than those observed in the GINA controlled asthma group. These findings may indicate the need for a more personalized management of patients with uncontrolled asthma, including intensification of counseling on the importance of smoking cessation-smoking being a modifiable risk factor and a factor contributing to the worsening of respiratory conditions-aggressive therapy for disease control; and close monitoring (allowing earlier intervention to minimize exacerbations and hospitalizations). On the other hand, these differences were not observed when the children were classified as having controlled or uncontrolled asthma by the C-ACT/ACT. Therefore, regarding disease control, the results of the present study suggest some superiority of the GINA questionnaire over the C-ACT/ACT. 

Regarding the level of asthma control as assessed by the GINA questionnaire and the C-ACT/ACT, Koolen et al.^6^ reported results that are similar to ours, although only 17% of the patients in their sample had controlled asthma as assessed by the GINA questionnaire and 40% had controlled asthma as assessed by the C-ACT/ACT. By comparing the results of the present study with those of that by Koolen et al.,^6^ we find similar sensitivity (72% vs. 66%, respectively) and specificity (100% in both studies). This suggests an overestimation of asthma control by caregivers and/or patients, an observation that has been reported elsewhere.^14^ ^-^ ^17^ With regard to the levels of asthma control as defined by the two questionnaires, we hypothesize that the cutoff for uncontrolled asthma on the C-ACT/ACT (i.e., 19 points) underestimates the proportion of children with uncontrolled asthma as assessed by the GINA questionnaire.^6^ Thus, the GINA questionnaire might be superior to the C-ACT/ACT for identifying obstructive ventilatory disorder, which has important clinical implications in the therapeutic management of these children. 

The GINA questionnaire assesses a broader range of factors using dichotomous questions regarding daytime and nighttime symptoms, as well as limitations in daily activities and use of relief medications, providing a more comprehensive view of asthma control.^18^ ^,^ ^19^ Despite having been validated and praised for its simplicity and ease of use, the ACT, through 4-5 possible answers to each question, one of which is subjective (question 5, which considers the judgment of control), focuses primarily on symptoms and activity limitations, giving less attention to other important points of asthma control assessment, such as exacerbations.^20^

One point to highlight is that the GINA questionnaire may be associated with spirometric parameters and may provide important information on asthma severity,^21^ especially FEF_25-75%_. Cottini et al.^21^ found no association between FEF_25-75%_ and the ACT, a finding that is consistent with those of the present study. Yi et al.^22^ found that children with poorer asthma control as assessed by the GINA questionnaire had worse FEV_1_ and FEF_25-75%_, a finding that is also consistent with those of the present study. 

It is known that higher doses of medications are needed to control asthma in patients with worse clinical severity.^2^ However, ours is the first study to show the superiority of the GINA questionnaire over the C-ACT/ACT in terms of ICS doses and disease severity in relation to the level of disease control. Nevertheless, previous studies^23^ ^,^ ^24^ highlighted some of the positive aspects of the GINA questionnaire used in combination with spirometric data for the management of pediatric asthma in terms of dose adjustment and assessment of asthma severity. 

The results of the present study show that the patients in the GINA controlled asthma group mainly used dry powder inhalers, whereas those in the GINA uncontrolled asthma group mainly used metered dose inhalers. Choosing an inhaler device is challenging because the choice must be based on patient preference and their ability to use the device, incorrect inhaler use resulting in poor lung deposition of the drug.^25^ ^,^ ^26^ Lavorini et al.^27^ found that patients had a greater preference for dry powder inhalers and that dry powder inhalers generally provide better pulmonary deposition of the drug than do other types of inhaler devices, a finding that might explain those of the present study. 

In addition to personal preference, the use of metered dose inhalers in children requires spacers (with or without a mask), which are not needed when dry powder inhalers are used. This can be a limiting factor because the inhaler device can be misplaced or inadequately cleaned, contributing to medication deposition on the spacer wall and having less medication available for pulmonary deposition.^28^ ^,^ ^29^ It is also important to consider the availability of medications in the Brazilian public health system. The medications may not always be the most suitable for patients, and the range of medications available in the public health system is not as wide as that available in the private health system. The patients in the present study were all recruited from among those followed at a public outpatient clinic, and the formoterol-budesonide combination (LABA+ICS) is often available only in inhaled capsule form. However, not all patients know how to use it correctly; therefore, treatment must be individualized with medications delivered in spray form, such as beclomethasone, which do not have the same bronchoprotective effect as do those in the LABA-ICS combination.^30^ ^,^ ^31^

In the present study, passive smoking showed a significant difference between the GINA controlled asthma and uncontrolled asthma groups, with a higher proportion of uncontrolled asthma among those exposed to passive smoking. Tobacco exposure is known to have a variety of harmful effects on children; it aggravates asthma symptoms, worsens asthma control, reduces lung capacity, and directly impacts quality of life.^32^ ^,^ ^33^ The literature corroborates our findings regarding loss of lung function in children exposed to smoking. Moshammer et al.^34^ showed an association between maternal smoking, reduced FEV_1_, and small airway obstruction, as evidenced by reduced FEF_25-75%_. McEvoy et al.^35^ demonstrated persistent deficits in lung function in children whose mothers smoked during pregnancy, including increased airway smooth muscle, collagen deposition, airway hyperresponsiveness, and reductions in forced expiratory flows persisting up to the age of 21 years. 

One of the limitations of the present study is that recruitment was carried out in a single center in Brazil among children with a specific socioeconomic profile (i.e., users of the public health system). Further studies investigating children with controlled and uncontrolled asthma should add a longitudinal follow-up to identify other factors related to the questionnaires used in the present study, as well as to study their responsiveness. 

In conclusion, although the GINA questionnaire and the C-ACT/ACT are universally accepted as tools for assessing asthma control, the GINA questionnaire proved to be more suitable for the children in the present study and showed a stronger association with functional parameters, indicating better clinical assessment with less underreporting of uncontrolled asthma. It is worth emphasizing the importance of health care professionals exercising caution when interpreting patient symptoms to avoid overestimating the clinical control of the disease. This ensures that the necessary therapeutic adjustments, including medication adjustments, are made for each patient. 
